# A web-based psychological support program for caregivers of children with cystic fibrosis: a pilot study

**DOI:** 10.1186/s12955-015-0211-y

**Published:** 2015-02-04

**Authors:** Astrid Fidika, Marion Herle, Christine Lehmann, Christa Weiss, Christine Knaevelsrud, Lutz Goldbeck

**Affiliations:** Department of Child and Adolescent Psychiatry/Psychotherapy, University of Ulm, Medical Centre, Steinhoevelstr. 1, Ulm, 89075 Germany; Department of Paediatric Pulmonology/Immunology, Charité, Humboldt University, Berlin, Germany; Department of Clinical Psychology and Psychotherapy, Freie University Berlin, Berlin, Germany

**Keywords:** Cognitive-behavioral therapy, Cystic fibrosis, Parents, Web-based intervention

## Abstract

**Background:**

Parents caring for a child with Cystic Fibrosis (CF) are at high risk for psychological distress and have limited access to psychological care. Therefore, a web-based psychological support program for severely distressed parents of children with CF (WEP-CARE) was developed and evaluated for its feasibility and efficacy.

**Methods:**

A clinical expert panel developed WEP-CARE based on principles of cognitive-behavioral therapy. This web-based writing therapy comprises nine sessions, tailored for the specific needs of caregivers. The pilot study was conducted as a single-group intervention with pre-post-follow-up design. Out of 31 participants, 23 parents completed the intervention (21 female; mean age 37 years; *SD* = 6.2 years, range 25 – 48 years). Psychological symptoms and quality of life were assessed online by self-report measures at pre- and post-treatment and were followed up three months later.

**Results:**

On average, the caregivers’ symptoms of anxiety decreased statistically significant and clinical relevant about five points from an elevated (*M* = 11.4; *SD* =2.6) to a normal level (*M* = 6.7; *SD* = 2.6; *p* < .001) between pre and post treatment. Fear of disease progression (*p* < .001) and symptoms of depression (*p* = .02) significantly decreased as well. Quality of life significantly improved (*p* = .01). The effects were maintained at the 3-months follow-up assessment.

**Conclusions:**

WEP-CARE is feasible and promising regarding its efficacy to improve parental mental health and quality of life.

## Background

Cystic Fibrosis (CF) is the most common inherited chronic and life limiting condition among Caucasians [1] with estimated between 70,000 and 100,000 patients worldwide [2]. Due to advances in diagnoses and clinical care, the great majority of the affected children nowadays survive into adulthood [3,4]. Nevertheless, CF as progressive and multi-organ disease impacts the functioning of the lungs and the pancreas, and requires an extensive and time-consuming therapy. Maintaining the daily disease-management is a challenge and coping with disease progression puts a high burden on the patients themselves and especially on their caregivers. Although the majority of caregivers cope well with their child's disease, parents of children with chronic conditions are known to be at high risk for developing psychosocial problems or disorders [5] and for impairments in their quality of life [6]. There is also substantial evidence of impaired life satisfaction and of a high prevalence of symptoms of anxiety and depression among caregivers of children with CF [7-9]. Within the context of chronic conditions parents are confronted with real threats, which might be overwhelming and may lead to difficulties to cope with everyday life and problems in the management of their child’s treatment routines. Fear of disease progression is known to be a relevant cause of distress in patients with chronic somatic conditions or their partners [10]. It is likely an issue for parental caregivers as well. In the long term the caregivers' illness-related distress may impair adherence to treatment [8] and thus result in worse disease outcomes in the child [7].

Although there is some evidence for especially cognitive-behavioral and problem solving interventions for parents of children with chronic conditions [11] studies investigating interventions for parents of children with CF are scarce [12]. Although the European consensus about standards of care in patients with cystic fibrosis claims integrated psychosocial care in CF centers [13,14], just a fractional amount of parents with clinically relevant symptoms of anxiety and/or depression received some kind of mental health care in a German study [15], indicating limited utilization and access to integrated psychosocial care. Du to unresolved reimbursement problems only a minority of German CF centers are able to directly offer psychological interventions to caregivers, and referrals to mental health services are impeded by long waiting lists. Barriers to caregivers' treatment comprise long distances to the CF center, restricted time capacities to care for themselves due to the demanding management of their child’s disease, or less awareness of the own well-being compared to the well-being of the child.

A wide range of studies on several mental disorders indicate that web-based interventions are efficacious and effective opportunities to overcome the mentioned barriers [16,17]. Research on web-based interventions in the context of pediatric chronic conditions is increasing. Studies on asthma or diabetes focused on optimization of disease management or on the patients themselves [18-20]. Only few studies addressed caregivers. Some of those studies examined online peer support, for example for parents of children with asthma and allergies [21], or educational programs to support parents following pediatric injury [22], or web-based problem solving intervention for families with children with traumatic brain injury [23]. However, no study so far investigated a web-based psychotherapeutic intervention solely for caregivers to address parental distress, related to the chronic condition of their child.

Due to the relevance of accessible psychotherapeutic support for caregivers of children with chronic conditions, the aim of the current study was to develop a web-based intervention for distressed caregivers of minors with CF which enables them to better cope with fears and demands of their child’s illness and evaluate it within a pilot study. We focused on the following research questions: First, we examined whether a web-based cognitive-behavioral writing therapy is feasible. Secondly, we investigated whether the intervention is efficacious to reduce symptoms of anxiety from pre-treatment to post-treatment (primary outcome). Furthermore, we expected that fear of disease progression and symptoms of depression would also decrease, parental quality of life (PQoL), as well as effective parental coping would increase. At least, we hypothesized that the benefit of treatment would persist until three months after completion of the intervention.

## Materials and methods

### Trial design

This study was conducted as a single-group intervention study. Primary outcome were symptoms of anxiety. The study protocol was approved by the Institutional Review Board (IRB) of the University of Ulm. All participants provided their written informed consent.

### Participants and procedure

The study was conducted through a secure internet platform (https://muko-wep.ulmer-onlineklinik.de/). Participants were recruited over a period of six months (June 2012 to December 2012) by various ways of distribution of information. In collaborating German speaking CF centres, flyers were distributed during routine visits. Information about the study was published in a member magazine, on the Facebook page and send via mailing lists of the German Cystic Fibrosis Association.

Eligible were caregivers of a child with CF (0–17 years) with clinically relevant symptoms of anxiety (≥8) on the anxiety subscale of the Hospital Anxiety and Depression Scale [24], being fluent in written German language, and having access to the internet during the intervention. Parents needed to be free of psychotic symptoms and suicidality. Parents who reported one of those symptoms were immediately contacted by the PI of the study to clarify symptoms and indication for psychiatric care. Parents who reported starting or changing of psychotropic medication about six weeks prior or during the intervention period, as well starting a competitive psychotherapy were excluded from the analysis.

Interested parental caregivers could register on the study website and then completed questionnaires. Parents turning out to be eligible for the study were asked to provide their written informed consent via mail. Parents who did not qualify for the study received a message recommending other opportunities to receive support.

### Intervention

The web-based psychological support program for caregivers (WEP-CARE) was developed by a clinical expert panel, based on previous studies [9,15,25,26] and a review of the literature on families coping with the CF of a child. The panel comprised clinicians with expertise in psychological counselling of patients with CF and their caregivers, and a clinician-researcher with expertise in internet-therapy. It is designed as web-based cognitive-behavioral writing therapy with three treatment components addressing the following disease-related aspects considered as relevant for caregivers of a child with CF: 1) exposure to thoughts associated with anxiety and enhance cognitive coping with the disease, especially with fears of disease progression, 2) sharing responsibility for the treatment of the child, and 3) increasing self-care. Once a week, one writing assignment should be completed within a timely standard of dealing approximately 45 minutes with the topic. The caregivers provided their written responses to nine standardized writing assignments and received individualized feedback to each delivery by their therapist within 48 hours. Thus, the communication of participants and interventionists was asynchronous all the time. Details of the treatment components and the corresponding writing assignments are summarized in Table 1.

**Table 1:** **Content of the intervention program**

**Treatment component**	**Writing assignment and its aims and content**
Preparation	Scheduling and providing potential dates for conducting the writing sessions.
	#1 – Reflection of current appraisal of their child’s CF and identification of desired changes in the impact of the condition upon their life using a metaphor.
Coping with anxiety and fear of disease progression	#2 – Selection of an anxiety-provoking situation or cognition and describing specifically the focus of their fear and their associated thoughts and feelings.
	#3 – Restructure own fear-provoking thoughts via writing a letter to a friend in a similar situation and thus to help their friend to develop a new perspective of the situation and to think about it in a more functional way.
	#4 – Intensification via a second letter going into details regarding strategies for their friend to gain a more adaptive and accurate perspective of the situation.
	#5 – Development of a strategy how to deal with the most threatening situation, if it actually occurs.
Sharing responsibility for treatment	#6 – Reflection on the allocation of treatment-related tasks within the family and if desired planning reallocation.
	#7 – Report of status of implementation of reallocation of treatment-related tasks and if not successful identification of barriers and development of strategies to deal with these barriers in the future.
Providing self-care	#8 – Learn to appraise positive things in life and caring for oneself. Identification of own needs via using a diary for positive experiences each day and planning enjoyable activities.
Summary and integration	#9 – Reflection and integration of the therapy contents and aims at preventing relapses via a letter to oneself.

The intervention program was provided by two trained and supervised psychotherapists with expertise in psychosocial care for patients with CF and their families.

Usability testing of WEP-CARE was conducted with three caregivers. The treatment manual is available from the authors on request.

### Outcomes

Demographic and medical information were collected at baseline. All other questionnaires were administered at baseline, after completion of the last treatment session, and three months after completion of the program.

#### Demographic and medical information

The following socio-demographic and medical data were collected by a self-constructed questionnaire: caregivers’ age, gender, native language, education level, employment status, living situation, experiences with the internet, and recently received psychological care, as well as the child’s age, gender, and the following health-related variables: pulmonary function (FEV_1_%), CF-related diabetes, haemoptysis, pneumothorax, chronic colonization with germs, liver problems, and being on a waiting list for a transplantation.

#### Symptoms of anxiety

Symptoms of anxiety were assessed with the anxiety subscale of the German version of the Hospital Anxiety and Depression Scale (HADS) [24]. The HADS is an easy-to-administer and brief screening measure used for assessing symptoms of anxiety and depression on two subscales (seven items each scale). On a four-point rating scale, ranging from zero to three, patients indicate the extent of every symptom with regard to the last seven days. For clinical discrimination of symptoms the following established cut-offs for the raw scores of each scale were used: normal (0–7 points), borderline (8–10 points), and clinical level (>11 points). The HADS is valid for using it in clinical as well as in community settings and has good psychometric properties [24].

#### Symptoms of depression

The short form of the German version of the Center of Epidemiologic Studies Depression Scale (CES-D) [27] was administered to assess following major components of depression: depressive mood, feelings of guilt and worthlessness, loss of appetite, and sleep disturbances. It is a 15-item self-report screening instrument for symptoms of depression. The On a 4-point scale (0 to 3) the frequency of symptoms are rated with regard to the last week. A total score is calculated (range 0 to 45). Psychometric properties are reported as excellent [27]. *Cronbach’s* alpha for the CES-D was appropriate for the use of the instrument in the study sample (*α* = .82).

#### Fear of disease progression

The Fear of Progression questionnaire for caregivers of youths with CF (FoP-Q-C) consists of 16 items, which were derived from existing versions of the questionnaire for patients with cancer and their partners [10,26] and adapted for the current study population based on interviews with clinical experts in family-oriented psychosocial care. The FoP questionnaires are judged as reliable and valid measures for the use in research and clinical care [10,28]. All items of the FoP-Q-C are answered on a five-point rating scale (1 to 5) and summed up to a total score (range 16–80). Psychometric properties in the sample of 50 caregivers being interested in WEP-CARE revealed good psychometric properties [29]. Internal consistency in the current study sample was good (*α* = .88). Moderate positive correlations with anxiety (*r* = .51; *p* < .01) and depression (*r* = .57; *p* < .01) were found, as well negative moderate correlations to quality of life (*r* = −.54; *p* < .01).

#### Parental quality of life

The Ulm Quality of Life Inventory for Parents of chronically ill children [30] is a 29-item self-report questionnaire measuring parental quality of life (PQoL) and was specifically developed for parents of children with chronic conditions. Parents indicate their well-being and functioning for each item on a five-point rating scale (0 to 4) with regard to the past seven days. This instrument covers the domains physical/daily functioning, satisfaction with family, emotional stability, self-development, well-being, as well as a total PQoL score. Psychometric properties are good [30]. *Cronbach*’s alpha in the current study ranged between .74 and .88 for the subscales (except subscale wellbeing *α* = .47) and .87 for the total score. All raw scores were linearly transformed to 0 – 100 scales. Higher scores indicate higher PQoL.

#### Parental coping

The German version of the Coping Health Inventory for Parents (CHIP) [31] was used to assess parental coping as a collection of coping strategies being related with their child’s chronic condition. This self-report questionnaire comprises 45 coping behaviors. For each item parents indicate the subjective effectiveness (scales between 0 and 3) of the specific strategy in the past and currently in managing the chronic condition of the child. Total score, as well as three subscales, describing the following coping patterns are composed by summing up raw scores: (I) maintaining family integration, cooperation and an optimistic definition of the situation (FAM), (II) maintaining social support, self-esteem, and psychological stability (SUP) and (III) understanding the medical situation through communication with other parents and consultation with medical staff (MED). Psychometric properties have been shown to be good [31]. *Cronbach’s* alpha for the three subscales ranged from .76 to .83 and was .83 for the overall scale.

### Statistical considerations

#### Sample size

According to the a priori sample size calculation by G-power, a sample size of N = 19 was required to detect at least a moderate to large pre-post effect (*d* ≥ 0.7) on a significance level of 5% (2-tailed) with a statistical power of 80%. To account for potential drop-outs and losses to follow-up we aimed to enroll a minimum of 30 participants.

#### Statistical analyses

All data were analyzed descriptively. Regarding the feasibility of WEP-CARE, the numbers of parental caregivers being interested and enrolled as well as the rate of participants completing the intervention were analysed descriptively. Reasons for not completing the program were described. Furthermore, any problems to carry out the study in accordance with the treatment manual were described.

To examine the efficacy of the intervention for reduction of symptoms of anxiety in the completers of the intervention, a paired *t*-test was conducted. All additional analyses regarding the secondary outcomes were explorative. For all secondary outcomes the significance level was set at *α* = .05. Similarly to the analyses for the primary outcome, paired *t*-tests were performed with the total score of each measure, as well as the subscales. To examine the clinical relevance of the results Reliable Change Indicies (RCI) [32] as well as percentages of caregivers with improved, stable or deteriorated scores were computed. The persistence of the benefits of treatment was examined by paired *t*-tests comparing the scores at pre-intervention and at the follow-up assessment. Effect sizes (Cohen’s *d*) were calculated to determine the magnitude of change.

## Results

### Study sample and feasibility

Recruitment was terminated with achievement of the sample size. Altogether, during the six months of enrolment 64 parental caregivers registered on the study website. Of the 50 who completed the baseline assessment, 37 met all inclusion criteria. The thirteen parental caregivers not meeting inclusion criteria all scored below the cut-off (<8) of the HADS anxiety subscale. Of the remaining 37 parents, three parents did not send their informed consent statement, and three did not start with the intervention after they were contacted by their therapist. Reasons for not providing an informed consent or not starting the intervention, although an informed consent had been provided, are unknown.

**Figure 1:**
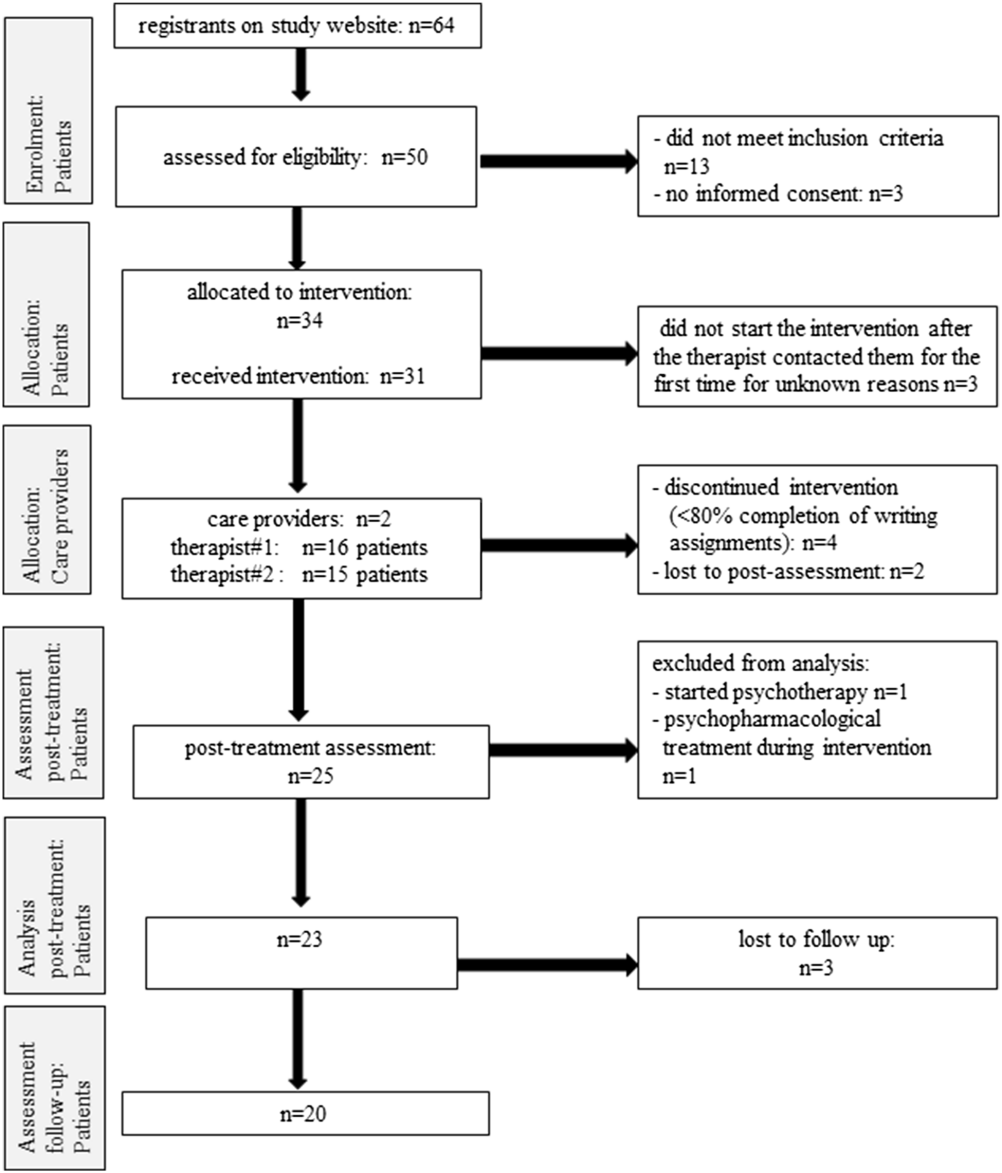
**WEP-CARE – CONSORT flow chart of the pilot study.**

Of the 31 parental caregivers starting with the intervention, four did not complete the program. Reasons for not completing the intervention were: having problems with the setting and the structure of the program (*n* = 1), health crisis of the child with CF (*n* = 1), and private issues not related to the child’s disease (*n* = 2). 27 of the participants (87.1%) were classified as completers of the intervention. Furthermore, two parents were lost to post-assessment and another two had to be excluded from the completer analyses, because they had started psychotherapy or psychopharmacological treatment during the intervention. All details of the progress of the participants through the study are summarized in Figure 1.

Of the 23 caregivers (mean age 37 years; *SD* = 6.2 years, range 25 – 48 years), who completed the intervention and were included in the post analyses, two were fathers (8.7%). Most participants (*n* = 20; 87%) were living together with their spouse or partner and (*n* = 15; 65.2%) reported to have graduated from high school. German was the native language of all participants. Further sample characteristics are presented in Table 2. Three of the participants had two children with CF (11.5%), all other one. Most of the children with CF were six years or younger (*n* = 18; 69.3%). More detailed information about the children with CF can be found in Table 3.

**Table 2:** **Characteristics of the study sample (n = 23)**

**Parental caregivers (** ***n*** **= 23)**		
Age		37.0 years (*SD* = 6.2; 25–48 years)
Gender	Male	2 (8.7%)
	Female	21 (91.3%)
Living situation	Living together with spouse/partner	20 (87.0%)
	Single parents	3 (13.0%)
Level of education	Secondary school	7 (30.4%)
	University entrance qualification	4 (17.4%)
	University degree	11 (47.8%)
	Other	1 (4.3%)
Employment status	Employed	19 (82.2%)
	Unemployed	4 (17.8%)

**Table 3:** **Characteristics of the children with CF (n = 26)**

**Children with CF (n = 26)**		
Age		5.87 years (*SD* = 4.8; 0–16 years)
Gender	Male	14 (53.8%)
	Female	12 (46.2%)
FEV_1_%	Unknown	16 (61.6%)
	<40%	0 (0%)
	40-70%	1 (3.8%)
	>70%	9 (34.6%)
CF related diabetes		0 (0%)
Chronic colonization with germs		5 (19.3%)
Liver problems		2 (7.6%)
Being on a waiting list for a transplant		0 (0%)

The intervention was performed throughout the pilot study in accordance to the treatment manual. The participants needed on average about three and a half months (*M* =114; *SD* = 37.4 days; range 68–222 days) from starting with the first writing assignment to completion of the intervention. About 75% of the participants completed the program within 3.2 months, but there were six persons who needed more time. The reason was postponing writing assignments because of unplanned disease-related tasks.

### Efficacy

All results for primary and secondary outcomes can be found in Table 4.

**Table 4:** **Results of the secondary endpoints of pre-post (n = 23) and 3-month follow-up (n = 20) comparisons**

	**Pre-post comparison (n = 23)**					**Pre-follow-up comparison (n = 20)**				
**Outcome**	***M*** **(** ***SD*** **) baseline**	***M*** **(** ***SD*** **) post**	***t (df)***	***p***	***d***	***M*** **(** ***SD*** **) baseline**	***M*** **(** ***SD*** **) follow-up**	***t (df)***	***p***	***d***
Symptoms of anxiety^1^	11.40 (2.6)	6.70 (2.6)	8.51 (22)	<.001	2.06	11.00 (2.38)	7.00 (3.40)	6.05 (19)	<.001	1.36
Fear of disease progression^2^	53.96 (11.50)	42.22 (9.60)	6.36 (22)	<.001	1.11	52.70 (11.66)	40.90 (10.96)	6.10 (19)	<.001	1.04
Depression^2^	16.96 (6.54)	12.09 (7.04)	2.48 (22)	.02	.72	16.60 (6.44)	11.65 (8.41)	2.34 (19)	.03	.66
Quality of life^2^_−_ Total score	49.73 (11.24)	57.88 (10.17)	−2.78 (22)	.01	.76	49.15 (11.33)	58.52 (14.46)	−2.57 (19)	.02	.72
Physical and daily functioning	53.55 (18.3)	58.98 (17.45)	−1.13 (22)	.27	.30	52.48 (17.39)	62.29 (20.01)	−2.37 (19)	.03	.52
Satisfaction with family	57.29 (19.07)	59.11 (18.12)	-.51 (22)	.62	.10	54.84 (18.51)	60.47(14.91)	−1.26 (19)	.22	.33
Emotional stability	43.21 (17.01)	64.67 (11.24)	−8.16 (22)	<.001	1.49	45.00 (16.76)	61.25 (19.30)	−3.99 (19)	.01	.90
Self-development	26.36 (17.27)	36.96 (16.85)	−2.46 (22)	.02	.62	26.88 (18.49)	36.56 (20.71)	−1.56 (19)	.14	.49
Well-being	57.34 (14.18)	68.21 (12.05)	−2.93 (22)	.01	.83	57.50 (13.39)	68.13 (17.07)	−2.29 (19)	.03	.69
Parental coping^2 –^ Total score	69.13 (21.22)	76.21 (16.78)	−1.79 (22)	.09	.37	70.15 (22.59)	70.10 (22.15)	.01 (19)	.63	.00
FAM	32.39 (10.01)	34.95 (9.00)	−1.27 (22)	.22	.27	32.40 (10.67)	33.25 (9.41)	-.49 (19)	.85	.08
SUP	24.91 (10.37)	29.34 (6.48)	−2.13 (22)	.04	.51	26.05 (10.53)	25.50 (9.91)	.19 (19)	.70	.08
MED	11.82 (5.13)	11.91 (5.59)	-.09 (22)	.93	.02	11.70 (5.07)	11.35 (5.74)	.39 (19)	.99	.06

*Annotation:* ^1^primary outcome; ^2^secondary outcome; effect size d: 0.2 small, 0.5 medium, and > 0.8 large effect; FAM: Maintaining family integration, cooperation and an optimistic definition of the situation, SUP: Maintaining social support, self-esteem, and psychological stability and III. Understanding the medical situation through communication with other parents and consultation with medical staff.

#### Primary endpoint

Before starting the intervention, the 23 parents scored on average at 11.4 (*SD* = 2.60) points on the anxiety subscale of the HADS. Symptoms of anxiety at post-treatment were significantly less (*t* = 8.51; *p* < .001) compared to pre-treatment. At post-treatment mean anxiety scores (*M* =6.70; *SD* =2.60) were below the cut-off for elevated symptoms of anxiety. The effect size was *d* = 2.06.

#### Secondary endpoints

Comparisons between pre- and post-treatment resulted in significantly lower fear of disease progression (*t* = 6.36; *p* < .001) and symptoms of depression (*t* = 2.48; *p* = .02). The effect size for fear of progression was *d* = 1.11 and for depression *d* = .72. After the program caregivers reported significantly higher PQoL on the total score (*t* = −2.78; *p* = .01), as well within the domains ‘emotional stability’ (*t* = −8.16; *p* < .001), ‘self-development’ (*t* = −2.46; *p* = .02), and ‘well-being’(*t* = −2.93; *p* = .01). Effect sizes ranged between *d =* .62 and 1.49. The scores on the sub-scale ‘social support, self-esteem, and psychological stability’ were significantly higher after the program (*t* = −2.13; *p* = .04). The effect size was *d* = .51. No further effects for coping could be found.

#### Clinical relevance of treatment effects

Details are shown in Table 5. A meaningful change regarding individual clinically significant reduction of symptoms of anxiety could be demonstrated for about 78% of the parents. Further, for about half of the parents a clinically relevant reduction of fear of disease progression, as well as for symptoms of depression was found. Nine parents (39%) showed clinically relevant improvements of their overall quality of life and most of them showed no relevant changes in their coping.

**Table 5:** **Clinical relevance of treatment effects**

	**RCI**	**Reduction** ***n*** **(%)**	**Steady** ***n*** **(%)**	**Increased** ***n*** **(%)**
Symptoms of anxiety	±3.03	18 (78.3%)	4 (17.4%)	1 (4.3%)
Fear of disease progression	±11.04	11 (47.8%)	12 (52.2%)	0
Symptoms of depression	±7.69	10 (43.5%)	11 (47.8%)	2 (8.7%)
	**RCI**	**Improved** ***n*** **(%)**	**Steady** ***n*** **(%)**	**Deteriorated** ***n*** **(%)**
Parental quality of life	±11.23	9 (39.1%)	11 (47.8%)	3 (13.1%)
Parental coping	±24.52	4 (17.4%)	18 (78.3%)	1 (4.3%)

#### Follow-up

The pre-follow-up comparison (*n* = 20) for anxiety revealed significant differences between baseline (*M* = 11.0; *SD* = 2.38) and three months after completion of the program (*M* =7.0; *SD* = 3.40; *t* = 6.05, *p* < .001), resulting in an effect size of *d* = 1.36.

Furthermore, paired *t*-tests revealed significant differences between pre-intervention assessment and three months follow-up for the following secondary outcomes: less fear of progression (*t* = 6.10; *p* < .001), decline in symptoms of depression (*t* = 2.34; *p* = .03) and increased overall PQoL (total score *t* = −2.57; *p* = .02), as well in the domains ‘emotional stability’ (*t* = −3.99; *p =* .01), ‘well-being’ (*t* = −2.29; *p =* .03) and ‘physical and daily functioning’ (*t* = −2.37; *p* = .03). No significant effects could be demonstrated for parental coping at the three-month follow-up.

## Conclusions

The current study aimed to evaluate a newly developed web-based cognitive-behavioral writing therapy for distressed caregivers of children and adolescents with CF. Altogether, the results confirm that web-based writing therapy is feasible for parents of children and adolescents with CF and efficacious to reduce distress and improve quality of life.

### Feasibility

The aimed recruitment rate of a minimum of 30 parents starting the intervention was achieved within a recruitment period of six months. The number of parents, who did not complete the program, was comparable to drop-out rates of face-to-face psychotherapy and equal to rates of completers reported for other web-based psychotherapeutic interventions [33]. Reasons for not completing varied and only one participant reported problems with the setting and the structure of the program. The time needed by the caregivers to pass through the whole program was of high range. The different duration for completion might be caused by intermediating time consuming demands due to the child’s illness as well as by the high workload and daily treatment routine in general [34]. Nevertheless, the treatment could be conducted in accordance to the treatment manual. Overall, our findings indicate that the program was feasible for the participants.

### Efficacy – symptoms of anxiety

Significant large and clinically relevant effects could be demonstrated on symptoms of anxiety at the end of intervention, as well as at three-month-follow-up. On average the caregivers’ symptoms of anxiety decreased 4.7 points on the anxiety subscale of the HADS from an elevated to a normal level. This finding is in line with previous studies on web-based CBT for anxiety [17,35,36] or PTSD [37].

### Efficacy -fear of disease progression, depression, quality of life, and coping

The results show that the caregivers’ fear of disease progression and symptoms of depression decreased, and parental quality of life increased between pre-intervention and post-intervention as well as between pre-intervention and 3-month follow-up. The significant reduction of both symptom scores indicates that participants as expected learned to cope with perceived fears and threats related to the illness of their child. Increase of parental quality of life in the domains ‘emotional stability’ and ‘self-development’ is consistent with the three main treatment components of the intervention and with the results of a systematic review on the application, clinical efficacy, and cost-effectiveness of internet-based cognitive behavioural therapy, demonstrating large effect sizes for internet-based treatments of depression, anxiety disorders, and health-related anxiety [16]. Regarding parental coping, just a trend for increase in effectiveness in coping strategies was found for the subscale ‘maintaining social support’ at post-treatment. Potential explanations are that the CHIP questionnaire [31] assesses general coping patterns in various situations rather than unique coping strategies and that there was just one treatment component explicitly addressing one of the three coping patterns assessed with the CHIP, namely ‘sharing responsibility for treatment’.

### Strengths and limitations

To our knowledge this is the first study evaluating a web-based psychological intervention for caregivers of a child with CF. However, some limitations should be mentioned. Due to the pilot character of this study a control group was lacking. We are not able to draw a conclusion whether the reported effects are specifically due to the participation on this intervention program. We did not investigate superiority of WEP-CARE to usual care, to the spontaneous course of psychological symptoms or to other active comparators.

The findings should be cautiously generalized to larger populations of caregivers due to the fact that mainly well-educated mothers were reached for participation. The requirement of written self-reflection might be associated to the level of education and literacy and therefore could be a barrier for participation for persons with a lower education. Gender diversity is often a problem in psychotherapy research, also in other studies on web-based interventions [37,38]. Moreover, as the lack of financial resources is an important risk factor for poorer health outcomes in pediatric respiratory diseases [39], it should be mentioned that the requirement of internet access could be an additional barrier for those families with low income. Limitations of self-report measures of psychological symptoms should be acknowledged.

### Implications for further research

Due to the preliminary results of our pilot study, WEP-CARE can be considered a promising approach to support parental caregivers of children and adolescents with CF. The findings can be taken as base for further efficacy research and dissemination of the intervention. Evidence for the specific efficacy and effectiveness can be aimed when conducting a randomized-controlled trial comparing to other psychological interventions, to usual care, or to no intervention.

As previously mentioned, distress and impairments of quality of life in caregivers of children with other pediatric chronic conditions is a well-known issue [6,40], which needs to be addressed. Transferability of the WEP-CARE for other rare and chronic diseases indicating the need to provide support to caregivers can be considered.

## References

[1] Littlewood JM, Hodson M, Geddes D, Bush A (2007). History of cystic fibrosis. Cystic Fibrosis.

[2] Cystic Fibrosis Worldwide (CFW) (2013). What is Cystic Fibrosis?.

[3] Dodge JA, Lewis PA, Stanton M, Wilsher J (2007). Cystic fibrosis mortality and survival in the UK: 1947–2003. Eur Respir J.

[4] Cystic Fibrosis Foundation (2013). Cystic Fibrosis Foundation Patient Registry 2012 Annual Data Report.

[5] Cohen MS (1999). Families coping with childhood chronic illness: a research review. Fam Syst Health.

[6] Hatzmann J, Heymans HS, Carbonell A, van Praag BM, Grootenhuis MA (2008). Hidden consequences of success in pediatrics: parental health-related quality of life-results from the care project. Pediatrics.

[7] Driscoll KA, Bennett Johnson S, Barker D, Quittner AL, Deeb LC, Geller DE (2010). Risk factors associated with depressive symptoms in caregivers of children with Type 1 diabetes or cystic fibrosis. J Pediatr Psychol.

[8] Smith BA, Modi AC, Quittner AL, Wood BL (2010). Depressive symptoms in children with cystic fibrosis and parents and its effects on adherence to airway clearance. Pediatr Pulmonol.

[9] Besier T, Born A, Henrich G, Hinz A, Quittner AL, Goldbeck L (2011). Anxiety, depression, and life satisfaction in parents caring for children with cystic fibrosis. Pediatr Pulmonol.

[10] Zimmermann T, Herschbach P, Wessarges M, Heinrichs N (2011). Fear of progression in partners of chronically Ill patients. Behav Med.

[11] Eccleston C, Palermo TM, Fisher E, Law E (2012). Psychological interventions for parents of children and adolescents with chronic illness. Cochrane Database Syst Rev.

[12] Goldbeck L, Fidika A, Herle M, Quittner AL (2014). Psychological interventions for individuals with cystic fibrosis and their families. Cochrane Database Syst Rev.

[13] Kerem E, Conway S, Elborn S, Heijerman H (2005). Standards of care for patients with cystic fibrosis: a European consensus. J Cyst Fibros.

[14] Conway S, Balfour-Lynn IM, De Rijcke K, Drevinek P, Foweraker J, Havermans T (2014). European Cystic Fibrosis Society Standards of Care: framework for the cystic fibrosis centre. J Cyst Fibros.

[15] Besier T, Goldbeck L (2011). Anxiety and depression in adolescents with CF and their caregivers. J Cyst Fibros.

[16] Barak A, Hen L, Boniel-Nissim M, Shapira N (2008). A comprehensive review and a meta-analysis of the effectiveness of internet-based psychotherapeutic interventions. J Technol Hum Serv.

[17] Hedman E, Ljótsson B, Lindefors N (2012). Cognitive behavior therapy via the internet: a systematic review of applications, clinical efficacy and cost-effectiveness. Expert Rev Pharmacoecon Outcomes Res.

[18] Grey M, Whittemore R, Jeon S, Murphy K, Faulkner MS, Delamater A (2013). Internet psycho-education programs improve outcomes in youth with type 1 diabetes. Diabetes Care.

[19] Mulvaney SA, Rothman RL, Osborn CY, Lybarger C, Dietrich MS, Wallston KA (2011). Self-management problem solving for adolescents with type 1 diabetes: intervention processes associated with an Internet program. Patient Educ Couns.

[20] Meischke H, Lozano P, Zhou C, Garrison M, Christakis D (2011). Engagement in "My Child's Asthma", an interactive web-based pediatric asthma management intervention. Int J Med Inform.

[21] Stewart M, Letourneau N, Masuda JR, Anderson S, McGhan S (2011). Online solutions to support needs and preferences of parents of children with asthma and allergies. J Fam Nurs.

[22] Marsac ML, Hildenbrand AK, Kohser KL, Winston FK, Li Y, Kassam-Adams N (2013). Preventing posttraumatic stress following pediatric injury: a randomized controlled trial of a web-based psycho-educational intervention for parents. J Pediatr Psychol.

[23] Wade SL, Walz NC, Carey J, McMullen KM, Cass J, Mark E (2012). A randomized trial of teen online problem solving: efficacy in improving caregiver outcomes after brain injury. Health Psychol.

[24] Hermann-Lingen C, Buss U, Snaith RP (2011). HADS-D Hospital Anxiety and Depression Scale - German Version.

[25] West CA, Besier T, Borth-Bruhns T, Goldbeck L (2009). Effectiveness of a family-oriented rehabilitation program on the quality of life of parents of chronically Ill children. Klin Padiatr.

[26] Goldbeck L, Hölling I, Schlack R, West C, Besier T (2011). The impact of an inpatient family-oriented rehabilitation program on parent-reported psychological symptoms of chronically Ill children. Klin Padiatr.

[27] Hautzinger M, Bailer M (1993). *Allgemeine Depressionsskala*, 1st edition edn.

[28] Herschbach P, Berg P, Dankert A, Duran G, Engst-Hastreiter U, Waadt S (2005). Fear of progression in chronic diseases: psychometric properties of the fear of progression questionnaire. J Psychosom Res.

[29] Fidika A, Mai S, Herle M, Goldbeck L (2014). 262 Fear of progression questionnaire for caregivers of youth with cystic fibrosis (FoP-Q/C). J Cyst Fibros.

[30] Goldbeck L, Storck M (2002). Das Ulmer Lebensqualitäts-Inventar für Eltern chronisch kranker Kinder (ULQIE): Entwicklung und psychometrische Eigenschaften. [ULQIE: a quality-of-life inventory for parents of chronically ill children.]. Z Klin Psychol Psychother.

[31] McCubbin H, McCubbin M, Cauble A, Goldbeck L (2001). Fragebogen zur elterlichen Krankheitsbewältigung: coping Health Inventory for Parents (CHIP) - Deutsche Version. Kindheit Entwicklung.

[32] Jacobsen NS, Truax P (1991). Clinical significance: a statistical approach to defining meaningful change in psychotherapy research. J Consult Clin Psychol.

[33] Melville KM, Casey LM, Kavanagh DJ (2010). Dropout from internet-based treatment for psychological disorders. Br J Clin Psychol.

[34] Hafen GM, Kernen Y, De Halleux QM (2013). Time invested in the global respiratory care of cystic fibrosis paediatrics patients. Clin Respir J.

[35] Spek V, Cuijpers P, Cek I, Riper H, Keyzer J, Pop V (2007). Internet-based cognitive behaviour therapy for symptoms of depression and anxiety: a meta-analysis. Psychol Med.

[36] Andrews G, Cuijpers P, Craske MG, McEvoy P, Titov N (2010). Computer therapy for the anxiety and depressive disorders is effective, acceptable and practical health care: a meta-analysis. PLoS One.

[37] Knaevelsrud C, Maercker A (2007). Internet-based treatment for PTSD reduces distress and facilitates the development of a strong therapeutic alliance: a randomized controlled clinical trial. BMC Psychiatry.

[38] Andersson G (2009). Using the internet to provide cognitive behaviour therapy. Behav Res Ther.

[39] Watts K, Schechter M (2010). Origins of outcome disparities in pediatric respiratory disease. Pediatr Ann.

[40] Cousino MK, Hazen RA (2013). Parenting stress among caregivers of children with chronic illness: a systematic review. J Pediatr Psychol.

